# QTL mapping for low temperature germination in rapeseed

**DOI:** 10.1038/s41598-021-02912-w

**Published:** 2021-12-03

**Authors:** Jifeng Zhu, Weirong Wang, Meiyan Jiang, Liyong Yang, Xirong Zhou

**Affiliations:** Shanghai Academy of Agricultural Sciences, Shanghai, 201403 China

**Keywords:** Genetics, Plant sciences

## Abstract

Rapeseed, a major oil crop in the world, is easily affected by low-temperature stress. A low temperature delays seed germination and increases seedling mortality, adversely affecting rapeseed growth and production. In the present study, a tolerant cultivar (Huyou21) was crossed with a susceptible genotype (3429) to develop a mapping population consisting of 574 F_2_ progenies and elucidate the genetic mechanisms of seed germination under low temperatures. Two quantitative trait loci (QTL) for low-temperature germination (LTG) were detected, one on chromosome A09 (named *qLTGA9-1*) and the other on chromosome C01 (named *qLTGC1-1*), using the QTL-seq approach and confirmed via linkage analysis in the mapping population. Further, *qLTGA9-1* was mapped to a 341.86 kb interval between the SSR markers *Nys9A212* and *Nys9A215*. In this region, 69 genes including six specific genes with moderate or high effect function variants were identified based on the Ningyou7 genome sequence. Meanwhile, *qLTGC1-1* was mapped onto a 1.31 Mb interval between SSR markers *Nys1C96* and *Nys1C117*. In this region, 133 genes including five specific genes with moderate effect function variants were identified. These specific genes within the two QTL could be used for further studies on cold tolerance and as targets in rapeseed breeding programs.

## Introduction

Rapeseed (*Brassica napus* L.) is a major winter crop mainly cultivated in the Yangtze River Basin of China, with a sown area of 6.6 million hectares and annual production of 13.5 million tons^1,2^. However, the cultivation patterns gradually changed from transplanting into no-tillage direct seeding with improvement in technology and an increase in labor cost^3^. The late direct seeding area under rapeseed has continuously increased in recent years, with intensive cropping development. Meanwhile, rapeseed is sensitive to temperatures below 10 °C at germination^4,5^. The temperature at late sowing time in China is generally below 10 °C, which leads to a low germination rate and seedling in rapeseed^6^, which ultimately affects yield^6,7^. Therefore, selecting varieties of rapeseed with a high rate of low-temperature germination (LTG) has become an important breeding goal under late direct seeding cultivation.

Different cultivars respond differently to low temperatures, and genetic factors largely control these differences. The LTG ability of crops is a complex trait controlled by quantitative trait loci (QTL)^6–8^. Several QTL mapping studies for LTG have been conducted in crops, such as rice, maize, soybean, and wheat^9–15^. Studies have found wide genotypic variation in LTG in rapeseed^16,17^ and associated a few QTL with low-temperature stress during seed germination and seedling stage. Xian et al.^6^ identified several differentially expressed genes related to low-temperature tolerance in rapeseed through transcriptome analysis. Luo et al.^7^ detected 22 QTL associated with low-temperature tolerance during seed germination and seedling stage through genome-wide association study (GWAS). However, genetic study on rapeseed germination under low temperature is still rare. Moreover, only a few markers associated with LTG have been developed for rapeseed breeding. Thus, it is critical to identify novel LTG QTL for fine mapping to accelerate the breeding of low-temperature tolerant varieties of rapeseed.

QTL associated with specific traits can be identified through different QTL mapping approaches, including QTL-seq. QTL-seq is an effective approach performed via bulked segregant analysis (BSA) using next-generation sequencing (NGS)^18^. QTL-seq has been used to rapidly identify QTL for different traits, such as spikelet fertility under heat stress in rice^19^, plant height in wild soybean^20^, and cold tolerance in wild rice^21^. The present study employed the F_2:3_ populations, derived from two inbred lines, Huyou21 (tolerant to low-temperature stress) and 3429 (susceptible to low-temperature stress), to detect the QTL related to rapeseed LTG via QTL-seq. The objectives of this study were to: (i) analyze seed germination of this population under low temperature; (ii) identify QTL for LGT from this population; and (iii) develop simple sequence repeat (SSR) markers of the LTG QTL for further fine-mapping or molecular breeding.

## Results

### Analysis of LTG and construction of bulks

**Figure 1:**
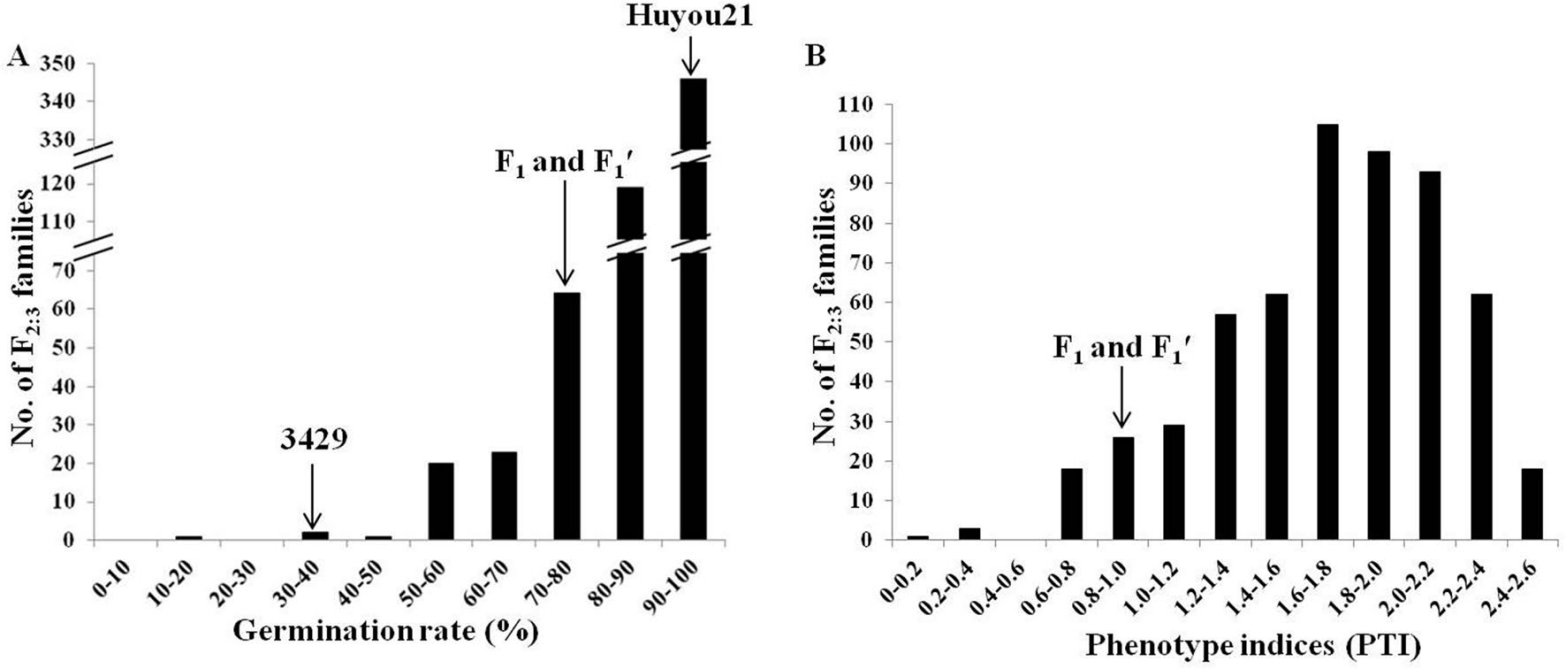
Distribution of the LTG phenotype in F_2:3_ population four days after imbibition (DAI) at 8 ℃.

Based on a preliminary screening for LTG, the tolerant cultivar Huyou21 and the susceptible cultivar 3429 were selected for further study. At optimal temperature (20 °C), the two parents demonstrated similar germination rates (> 95%) within four days after imbibition (DAI). At low temperature (8 °C), Huyou21 exhibited excellent tolerance to cold stress, with a mean germination rate higher than 90% within four DAI. In contrast, 3429 showed high susceptibility at 8 °C, with a mean germination rate lower than 40% within four DAI (Fig. 1A). The mean germination rate of all F_1:2_ seeds derived from the F_1_ plants of 3429 × Huyou21 cross was 76.9%, and that derived from the F_1_′ plants of Huyou21 × 3429 cross was 70.2%. The F_2_ lines showed large germination rate variations, ranging from 17.5% to 100.0%, exhibiting a distribution skewed toward tolerance (Fig. 1A).

Regression analysis of parent–offspring revealed an LTG heritability estimate of 0.26 for the F_2:3_ families. After cold stress, 344 plants out of F_2:3_ families showed a germination rate larger than 90%, and 230 plants showed a germination rate equal to or less than 90%. Besides, the tolerant and susceptible plants fit well in the 9:7 ratio (*χ*^2^ = 1.58, *p* = 0.21) (Table S1). These results indicate the role of two dominant genes in controlling LTG in Huyou21. The F_2:3_ families showed pronounced variation and segregation in cold stress tolerance or sensitivity. The LTG tolerance distribution was continuous and approximately normal in the F_2:3_ families, indicating a quantitative inheritance of LTG. Meanwhile, the phenotypic trait indices (PTI) of F_2_ progeny ranged from 0.19 to 2.58 (Fig. 1B). To establish the low temperature tolerant (LT) bulk and low-temperature sensitive (LS) bulk, 30 LT and 30 LS F_2_ individuals were selected. The PTI of the 30 plants in the LT bulk was more than 2.33, and that of the 30 plants in the LS bulk was less than 0.99.

### Resequencing and mapping of reads

The two bulks (LT bulk and LS bulk) with extreme phenotypes (the tolerant and sensitive pools) and the two parents were used to prepare the libraries for Illumina sequencing. A total of 319,059,584 reads were generated for LT bulk, 335,343,844 reads for LS bulk, 323,983,156 reads for tolerant parent (Huyou21), and 337,990,498 reads for susceptible parent (3429). Alignment of the reads to the Ningyou7 rapeseed genome sequence^22^ revealed 36.52 × and 37.58 × read depth and 99.17% and 99.13% coverage for LT bulk and LS bulk, respectively. Similarly, 36.41 × and 38.26 × read depth and 95.73% and 97.31% coverage were obtained for Huyou21 and 3429, respectively (Table 1). In total, 5,600,575 genome-wide SNPs and 1,215,792 InDels were obtained for LT bulk, and 5,604,534 SNPs and 1,217,972 InDels for LS bulk by comparing with the reference genome (Table S2 and Fig. S1).

**Table 1:** Summary of the sequencing results for the parental lines and two bulks.

Genotypes	Clean reads	Clean base (Gb)	Read alignment (%)	Average depth ( ×)
LT bulk	319,059,584	47.48	99.17%	36.52
LS bulk	335,313,844	49.89	99.13%	37.58
Huyou21	323,983,156	48.17	95.73%	36.41
3429	337,990,498	50.36	97.31%	38.26

### Candidate genomic regions identified for LTG by QTL-seq

**Figure 2:**
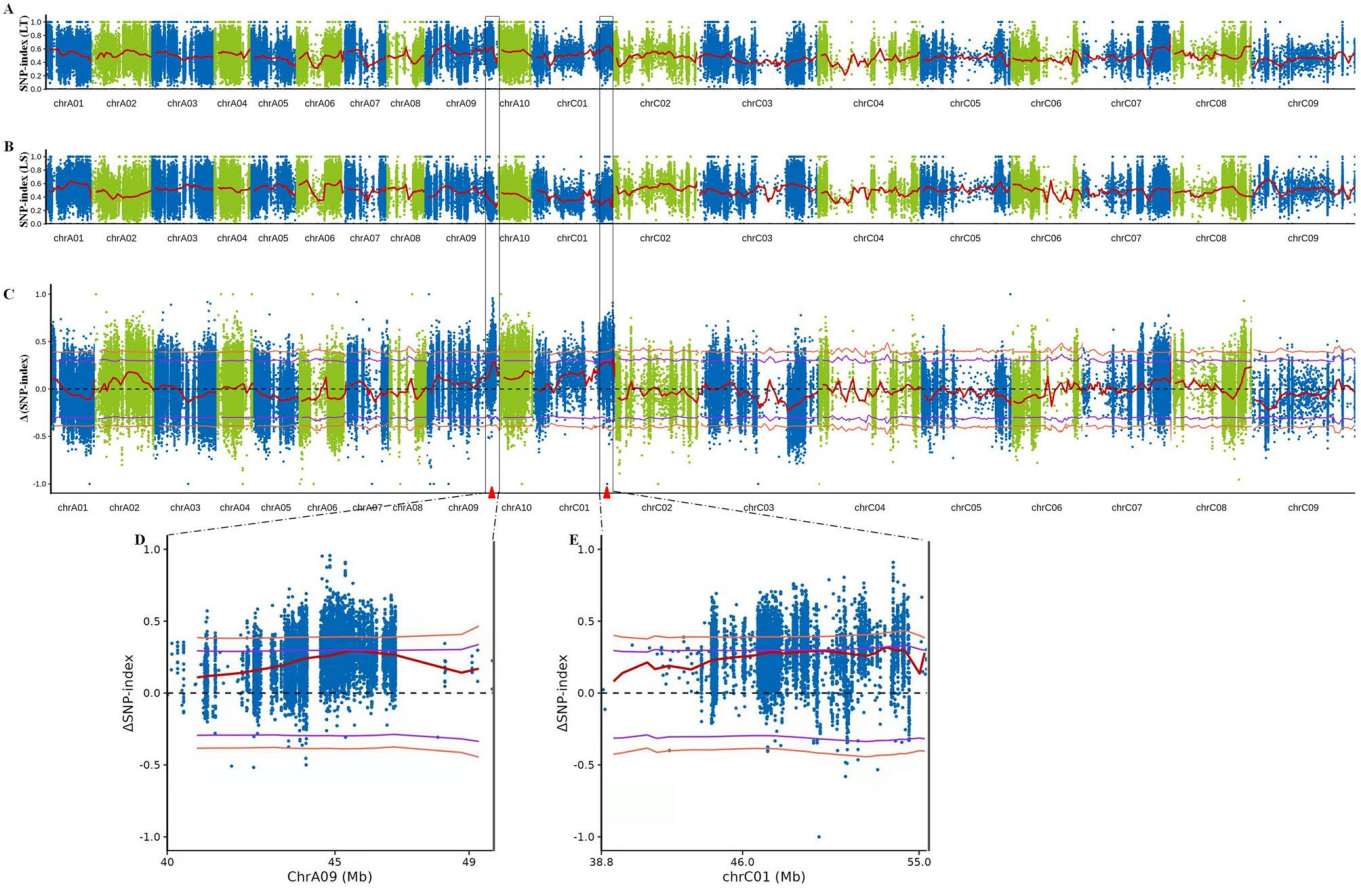
Identification of LTG QTL in F_2_ populations by QTL-seq. (**A**, **B**, and **C**) represent the SNP-index plots of LT bulk and LS bulk, and the Δ(SNP-index) plot of 19 rapeseed chromosomes from QTL-seq analysis, respectively. Δ(SNP-index) plot with statistical confidence interval under the null hypothesis of no QTL (purple, *P* < 0.05; orange, *P* < 0.01). (**D**)and (**E**) represent the significant genomic regions of 40.00–49.73 Mb on chromosome A09 and 38.90–55.38 Mb on chromosome C01 identified for LTG, respectively. The X-axis represents the chromosomes of rapeseed, and Y-axis represents the SNP-index.

Further, the SNP index was calculated for each bulk to identify the candidate genomic regions related to LTG. Then, Δ(SNP-index) was calculated and plotted against the genome positions by combining the information on the SNP-index in LT bulk and LS bulk (Fig. 2). The Δ(SNP-index) was calculated by subtracting the SNP-index of LS bulk from the SNP-index of LT bulk, and plotted across the 19 rapeseed chromosomes to map the putative genomic regions associated with the phenotype for LTG. Two QTL regions on the two chromosomes A09 and C01 were detected based on Δ(SNP-index) plot (Fig. 2C), at 95% significance. The peak of *qLTGA9-1* was located between 40.00 Mb and 49.73 Mb on chromosome A09 (Fig. 2D), and that of *qLTGC1-1* was located between 38.90 Mb and 55.38 Mb on chromosome C01 (Fig. 2E). The results revealed one QTL related to LTG at the 9.73 Mb region of chromosome A09, named *qLTGA9-1*, and another QTL at the 16.48 Mb region of chromosome C01, named *qLTGC1-1*.

### Marker development and QTL fine mapping

**Figure 3:**
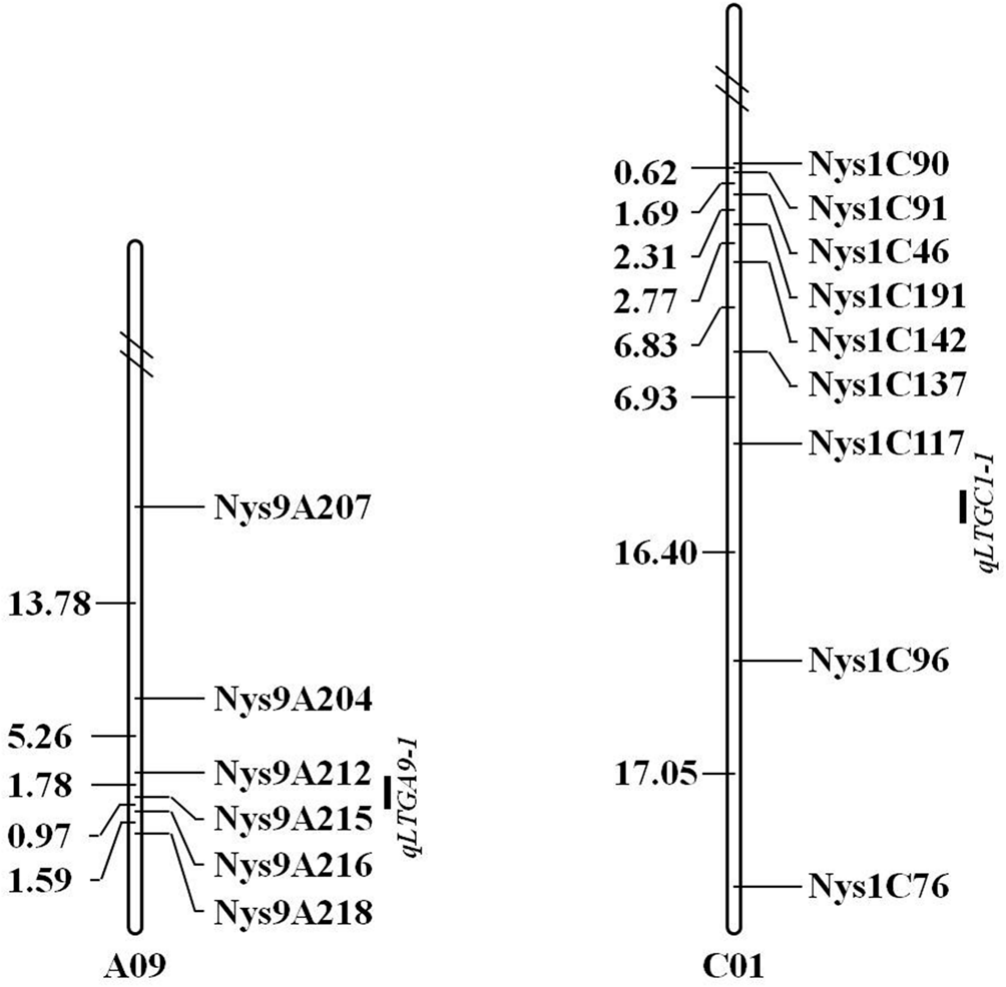
Fine mapping of LTG QTL in F_2:3_ families derived from 3429 × Huyou21. The numbers on the left are map interval sizes in Kosambi centiMorgan (cM) units and on the right are the SSR markers on chromosome A09 and C01.

A total of 351 SSR markers, including 163 primers in the 9.73 Mb candidate region (*qLTGA9-1*) on chromosome A09 and 188 primers in the 16.48 Mb candidate region (*qLTGC1-1*) on C01, were developed and genotyped in Huyou21 and 3429 to analyze the result of QTL-seq. Among these, 17 primer pairs (eight on A09 and nine on C01) produced steady and clear polymorphic bands between the two parents (Table 2), indicating that these primers could be used in QTL mapping for LTG. These polymorphic markers were selected for linkage map construction, and linkage analysis was performed in the 574 F_2_ individuals with the LTG phenotype. The genetic map and the LTG phenotypic analysis demonstrated both QTL on the predicted regions (Fig. 3). The QTL *qLTGA9-1* was mapped in a 1.78 cM interval between the tightly linked markers, *Nys9A212* and *Nys9A215*, which only explained 5.93% of the phenotypic variation for LTG but had a higher Limit of detection (LOD) value of 8.43 (Fig. 3 and Table 3). Meanwhile, *qLTGC1-1* was mapped in a 16.40 cM interval between the tightly linked markers, *Nys1C96* and *Nys1C117*, which explained a phenotypic variation of 5.39% and had a LOD value of 6.57 (Fig. 3 and Table 3).

**Table 2:** Polymorphic markers used for linkage analysis.

Markers	Primer sequences		Chr^a^	Location^b^
	Forward (5′-3′)	Reverse (5′-3′)		
Nys9A204	GCATGTATAACTCCTTGAATCC	CCACAAGAAATTAGAGGTCG	A09	43716935–43717137
Nys9A207	TACTCCTTTGGAAGGAAACA	TCCCTCTCCATCTGAAAATA	A09	44059061–44059257
Nys9A208	ATTCTGTGTATCCCATTTCG	GAGGAGGTTTCTGAGGAGTT	A09	44230061–44230241
Nys9A145	TTCAGTAAAGCTGACACACG	CGAGAGGTTATTAGGGGTTT	A09	44611834–44612080
Nys9A212	CAAAAGAGGGAATTTCAGTG	TGTCTCTAGTGAGAAAGCATTG	A09	44721694–44721885
Nys9A215	AACACACAGACATCGAGACA	TGAGATTGAAAGAGAAGGGA	A09	45063742–45063891
Nys9A216	CCCTGGATATAGCTTGTGAT	AGACCTTTTTCATTGTCAGG	A09	45165658–45165884
Nys9A218	TCTGGACAGCATCTTTAGGT	CTCAGACAACTCAGCAATCA	A09	45389536–45389768
Nys1C90	ATAAATAGCCCCACGTCTCT	ACAGAGGAAATCGAAACAGA	C01	47748257–47748503
Nys1C91	GGGAGGTTCTAGGACAAAAT	AGTGACAGATGGGATCAAAC	C01	47788771–47789026
Nys1C96	GAGCTTAAGGGCTCTTCTTC	TAGGAGACTTGACCATCCAC	C01	48189600–48189816
Nys1C46	CTGAAACCCAAAAATAGCG	TAGATCCAGCTAAGTACCCG	C01	48690912–48691125
Nys1C191	CACGTACAATCAGTCAACCA	CCATCACCATGAAAATCTCT	C01	49158881–49159050
Nys1C117	CTGGATAAAAAGAAACGTGG	TCTCTTCTTCATGCCTCTGT	C01	49503123–49503320
Nys1C137	CTTTCTCCTCTTTTCCGATT	CCTTGCTTAGACTTCTTGGA	C01	51668433–51668607
Nys1C142	TCCTTACCGCAAGAAACTAC	CGACGAGGAGTATGAGTAGG	C01	52183392–52183583
Nys1C76	TTCTCTCTTGGGAAAAGTCA	AGCCTTTGAAGACTAAACCC	C01	54524191–54524377

^a^Chr is the chromosome.

^b^Location is the marker position on the reference genome sequence.

**Table 3:** Statistics of LTG QTL identified at four days after imbibition (DAI) in F_2:3_ families.

QTL	Chr^a^	Marker Interval	Distance (cM)^b^	LOD	PVE (%)^c^	Add^d^	Dom^e^
*qLTGA9-1*	A09	Nys9A212-Nys9A215	1.78	8.43	5.93	0.13	0.14
*qLTGC1-1*	C01	Nys1C96-Nys1C117	16.40	6.57	5.39	0.08	0.18

^a^Chr is the chromosome of rapeseed.

^b^Distance means the distance between the left and right marker of the mapping QTL.

^c^PVE represents the phenotypic variation of LTG in F_2:3_ families by each QTL.

^d^Add is the additive effect.

^e^Dom is the dominant effect.

Based on the physical position of the tightly linked markers, *qLTGA9-1* was localized in a 341.86 kb interval between 44.72 Mb and 45.06 Mb on rapeseed chromosome A09, and *qLTGC1-1* in the 1.31 Mb interval between 48.19 Mb and 49.50 Mb on chromosome C01. Further, annotation based on Ningyou7 genome sequence^22^ predicted that 69 and 133 genes were located in the *qLTGA9-1* region and *qLTGC1-1* region, respectively. Further, 74 SNP and 25 InDel mutations related to 32 predicted genes in the *qLTGA9-1* region, and 162 SNP and 26 InDel mutations related to 35 predicted genes in the *qLTGC1-1* region were screened based on the sequencing results for each QTL (Table S3 and Table 4). Of these predicted genes, only one gene in the *qLTGA9-1* region was a variant (frameshift insertion/deletion) with a high effect, and five genes in *qLTGA9-1* and *qLTGC1-1* regions were variants (missense mutation) with moderate effects (Tables 4 and 5). These gene variants with moderate or high effects may be related to low-temperature tolerance at the generation stage in rapeseed.

**Table 4:** Candidate genes located in the intervals of *qLTGA9-1* and *qLTGC1-1*.

QTL	QTL region (Mb)^a^	Number genes	Number genes with high effect variant^b^	Number genes with moderate effect variant^b^
*qLTGA9-1*	44.72–45.06	32	1	5
*qLTGC1-1*	48.19–49.50	35	0	5

^a^QTL region defined as union of QTL-seq and linkage mapping credible intervals.

^b^Functional effects of variants (moderate or high) determined by snpEff tool.

**Table 5:** Annotation of the candidate genes with moderate (high) effect variants in *qLTGA9-1* and *qLTGC1-1* regions.

QTL	Gene ID	Start base–end base (bp)^a^	Functional effect^b^	Chain	Predicted function^c^
*qLTGA9-1*	*ChrA09g005501*	44842926–44843909	Moderate	Forward	Formiminotransferase
	*ChrA09g005502*	44845212–44845795	Moderate	Reverse	–
	*ChrA09g005507*	44864501–44865259	Moderate	Forward	Protein phosphatase 2C
	*ChrA09g005509*	44869664–44570813	High	Forward	Aminotransferase class I and II
	*ChrA09g005523*	44950207–44952401	Moderate	Forward	DRG Family regulatory protein
	*ChrA09g005524*	44954947–44959392	Moderate	Forward	DRG Family regulatory protein
*qLTGC1-1*	*ChrC01g004357*	48588162–48594007	Moderate	Forward	SWEET sugar transporter
	*ChrC01g004359*	48596431–48597483	Moderate	Forward	PHD-finger
	*ChrC01g004400*	49078002–49082772	Moderate	Forward	Plant invertase/pectin methylesterase inhibitor
	*ChrC01g004405*	49166002–49166734	Moderate	Reverse	–
	*ChrC01g004406*	49193433–49194020	Moderate	Forward	–

^a^Physical position of predicted gene obtained from the reference genome of Ningyou7.

^b^Functional effects of variants (moderate or high) determined by snpEff tool.

^c^Predicted function of candidate gene based on Ningyou7 annotation information.

“–” indicates no putative conserved domains have been detected.

The Ningyou7 annotation^22^ identified that seven of the predicted genes (four genes in *qLTGA9-1* and three genes in *qLTGC1-1*) encode proteins associated with plant growth or temperature stress response as follows: *ChrA09g005501* encodes a formiminotransferase, *ChrA09g005507* encodes a protein phosphatase 2C (PP2C), *ChrA09g005509* encodes an aminotransferase, *ChrA09g005523* and *ChrA09g005524* encode DRG family regulatory proteins (DFRP), *ChrC01g004357* encodes a SWEET sugar transporter, *ChrC01g004359* encodes a PHD-finger, and *ChrC01g004400* encodes a plant invertase/pectin methylesterase inhibitor (PMEI) (Table 5).

## Discussion

The late direct seeding area for rapeseed in China has continuously increased with intensive cropping development. However, low temperature occurs in late-autumn or early-winter quickly affects rapeseed germination with the delay of rapeseed seeding times^6^. Therefore, it is a breeding goal to select varieties with good germination under both optimal and stressed conditions to adapt to changing temperatures under late direct seeding cultivation. In the previous studies, a high degree of variability was observed in the seed germination rate among the rapeseed genotypes under low-temperature conditions. The genotypic variability helped LTG studies in rapeseed breeding to deal with cold stress under late direct-seeding conditions.

Studies have also revealed variations in LTG among plant species, which are generally affected by inheritance and environmental factors and regulated by QTL and multi-genes^6–8^. QTL mapping studies for LTG have been conducted in various crops, such as rice, maize, soybean, and wheat^9–15^; however, few QTL or genes for LTG are reported in rapeseed. QTL-seq combined with BSA and NGS is a practical approach used to identify QTL^18^. The method has been successfully used to rapidly identify QTL of different traits in rapeseed^23,24^. In this research, a segregating population was employed to detect the LTG QTL of rapeseed using QTL-seq. The analysis of the LTG in the population derived from the 3429 × Huyou21 cross revealed that two dominant nuclear genes or QTL controlled the LTG of these populations, with a heritability of 0.26. Further, QTL-seq revealed two QTL (namely *qLTGA9-1* and *qLTGC1-1*) associated with LTG on chromosomes A09 and C01; both were verified with classical QTL analysis through map construction. Here, *qLTGA9-1* was mapped between the flanking SSR markers *Nys9A212* and *Nys9A215*, and *qLTGC1-1* was mapped between *Nys1C96* and *Nys1C117*. Besides, the study found that the QTL (*qLTGA9-1*) on chromosome A09 was mapped around 30.2 Mb apart from each other based on the physical position of linkage markers on ZS11 genome sequence^25^. Earlier, GWAS mapping by Luo et al.^7^ showed that the candidate genes related to seed vigor under low temperature were localized around the physical position of 3.0 Mb on chromosome A09 based on ZS11 genome sequence^25^. As the physical distance between the locus and the QTL identified in this study is more than 27 Mb, the QTL, including *qLTGC1-1*, identified are novel in rapeseed for LTG.

Furthermore, to recapitulate the physical position of the linkage markers (*Nys9A212* and *Nys9A215*, *Nys1C96* and *Nys1C117*) based on the Ningyou7 genome sequence^22^, 69 and 133 genes were predicted in *qLTGA9-1* and *qLTGC1-1*, respectively, and 11 of these predicted genes were variants with moderate/high effect according to QTL-seq. Among these predicted genes were *ChrA09g005507* in the *qLTGA9-1* region encoding a PP2C and *ChrC01g004357* in the *qLTGC1-1* region encoding a SWEET protein. PP2C is a key player in ABA signal transduction, which plays an important role in seed germination under cold stress^26–28^, while SWEET protein is an important plant sugar transporter family and plays a crucial role in seed germination and stress response^29,30^. In addition, *ChrA09g005523* and *ChrA09g005524* in *qLTGA9-1* region encode DFRPs, while *ChrC01g004359* and *ChrC01g004400* in *qLTGC1-1* region encode a PHD-finger and a PMEI, respectively. Like PP2C or SWEET, the DFRP, PHD-finger, and PMEI proteins also play significant roles in defense response or under abiotic stress^31–33^. However, further research should analyze and functionally validate these candidate genes.

## Materials and methods

### Plant materials

**Figure 4:**
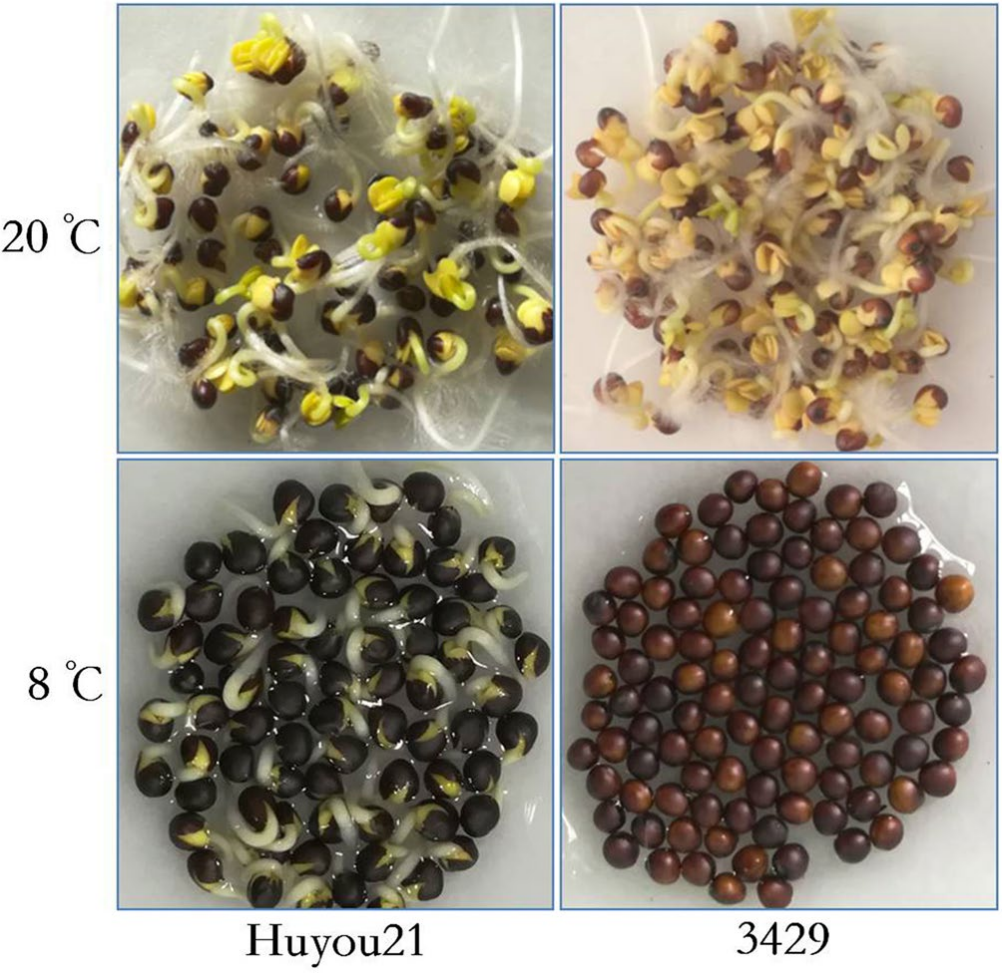
Huyou21 and 3429 germination four days after imbibition (DAI) under 20 ℃ and 8 ℃.

The parents used to develop the mapping population were Huyou21 (tolerant to cold stress) and 3429 (susceptible to cold stress) (Fig. 4). Seeds of Huyou21 and 3429 cultivars were obtained from the Shanghai Academy of Agricultural Sciences. Huyou21, developed from a double-cross between rapeseed lines 9714/9711 and 84004/8920, is widely cultivated in the lower reaches of the Yangtze River Basin, China, while 3429, used as the female, is a new line derived from a self-cross plant of the hybrid variety Qinyou99. The 3429 × Huyou21 hybrids were advanced from the F_1_ generation by selfing to yield the F_2:3_ families for mapping of LTG. Besides, the seed germination of 500 seeds harvested at the same time from both parents and their generations was confirmed.

### Phenotypic evaluation

Five hundred healthy and plump F_3_ seeds obtained per F_2_ plant, all together F_2_ seeds per F_1_ plant, and their parental controls were placed on two layers of filter paper with 15 mL distilled water in Petri dishes (9 cm inner diameter). The germination experiment was conducted in a low-temperature incubator set at 8 °C in a completely randomized design with three replications per treatment. The number of germinated seeds (*N*_*1*_) was counted on the fourth day after imbibition (DAI). Seeds that did not germinate after four days under low temperature (8 °C) were removed to normal temperature (20 °C) to start the recovery process for three days, and the number of remaining germinated seeds (*N*_*2*_) under normal temperature were counted to exclude the percentage of low-vigor seed influences. A seed was considered germinated once the radicle emerged and elongated 2–3 mm from the seed. The seed germination under low-temperature was calculated using the following formula:

$$ {\text{Germination\,rate}}\,(\% ) = N_{1} /\left( {N_{1} + N_{2} } \right) \times 100\% . $$

Plants with a germination rate larger than 90% were classified as cold tolerant, and those with a germination rate equal to or less than 90% were classified as cold susceptible. The broad-sense heritability of LTG expression was estimated using parent–offspring regression methods based on the variance of parents, F_1_-derived F_2_ population, and F_2_-derived F_3_ families^34^. The data on germination rate were arcsine transformed to improve the homogeneity of variance^35,36^. Further, the phenotypic trait indices (PTI) of F_2:3_ families for QTL mapping of LTG were calculated using the following formula to reduce the influence of microenvironment features during cold treatments:

$$ {\text{PTI}} = 2 \times \arcsin (X)/[\arcsin (P_{1} ) + \arcsin (P_{2} )], $$

where *X* represents the grand mean of germination rate of the F_2:3_ family, *P*_*1*_ and *P*_*2*_ represent the germination rate of their parental controls. The PTI of F_2:3_ families was evaluated and analyzed via chi-square test using SAS software (v9.1, SAS Institute, Cary, NC).

### Sample bulking and DNA isolation

A total of 574 F_2_ individuals from 3429 × Huyou21 cross were selected to build DNA bulks for QTL-seq. Thirty individuals for LT bulk and another 30 individuals for LS bulk were selected from the F_2_ population based on the extreme phenotype of F_2:3_ families for PTI under the low temperature (8 °C). Genomic DNA from LT bulk, LS bulk, F_2_ individuals and their parents (Huyou21 and 3429) were isolated using Plant Genomic DNA Kit (TIANGEN, China), following the instructions. DNA quality and concentration were examined by agarose gel electrophoresis (1%; w/v).

### Illumina sequencing and NGS data analysis

Test-qualified genomic DNA samples from LT bulk, LS bulk, and two parents were used to construct libraries with an insert size of 350–500 bp at Shanghai OE Biotech Co., Ltd. (China) using the TruSeq DNA LT Sample Prep Kit and sequenced (150 bp pair-end reads) using an Illumina Xten platform. Raw data generated from Illumina sequencing were subjected to quality control using Trimmomatic (v0.36)^37^. The filtered clean reads from both parents and two DNA bulks were aligned to the rapeseed genome sequence^22^ using BWA software, and single nucleotide polymorphism (SNP) calling was performed with SAMtools^38^. The average SNP-index for each pool was calculated in 1 Mb sliding windows with a 10 kb increment. The △(SNP-index) was calculated by subtracting the SNP-index of LS bulk from that of LT bulk. All SNP-index and △(SNP-index) were calculated for all positions as previously described^18,39^ to identify LTG-related QTL.

### SSR marker analysis and QTL fine mapping

LTG-related QTL identified by QTL-seq were validated and fine mapped through the traditional QTL mapping method^40^. A total of 351 SSR markers in the predicted regions were mined from the whole-genome sequence^22^. The SSR markers were used to survey the polymorphism between parents, which were designed with SSR Locator^41^ based on the parameters as previously described^40^. The newly developed markers were named *NysX(A/C)Y* markers, where *Nys* represents the microsatellite from the physical sequence of Ningyou7 rapeseed, the number *X* indicates the chromosome in subgenome (A or C), and *Y* represents a numerical code for the newly designed marker. Further, PCR and amplicon detection for the F_2_ individuals and their parents were performed as previously described^42^ with minor modifications. Polymorphic markers were further selected to analyze F_2_ population to construct a linkage map for QTL fine mapping. The genetic linkage map was drawn using the MAP functionality in QTL IciMapping v4.1^43^, and QTL was conducted using the BIP functionality^43^. The map distance (cM) was calculated using the Kosambi mapping function^44^, and the mapping method adopted was ICIM-ADD^45^. The LOD threshold and recombination frequency were set at 3.0 and 0.30, respectively. The QTL were designated by the term *qLTG* followed by the chromosome number.

### Candidate gene annotation

The candidate genes within the detected QTL for LTG were obtained based on the Ningyou7 rapeseed genome annotation^22^, and the functional variant effects of SNPs or Indels in each gene were predicted using SnpEff^46^ software.

### Permission statement

All the experiments on plants, including the collection of rapeseed materials, were performed in accordance with relevant guidelines and regulations.

## Conclusions

A total of 574 F_2:3_ families were constructed to elucidate the genetic mechanisms of seed germination under low temperature in rapeseed. Based on the QTL-seq and linkage analysis of the populations, two QTL were identified from ‘Huyou21’. One QTL was mapped to a 341.86 kb interval between the SSR markers *Nys9A212* and *Nys9A215* on rapeseed chromosome A09, and another was mapped to a 1.31 Mb interval between the SSR markers *Nys1C96* and *Nys1C117* on chromosome C01. These findings provide a basis for further studies on genetic breeding and assist in cloning candidate genes for cold tolerance in rapeseed.

## Supplementary Information

Supplementary Information.

## Data Availability

This whole genome resequencing reads used in QTL-seq has been deposited in the National Center of Biotechnology Information Sequence Read Archive (SRA) under BioProject accession number PRJNA751740.
