# Supplementary material for: QTL mapping for low temperature germination in rapeseed
Source: Sci Rep. 2021 Dec 3;11:23382. doi: 10.1038/s41598-021-02912-w (PMC8642550; doi:10.1038/s41598-021-02912-w)
Supplement: Supplementary file 1 — Supplementary Information. [file 41598_2021_2912_MOESM1_ESM.pdf]

QTL mapping for low temperature germination in rapeseed

Authors: Jifeng Zhu, Weirong Wang, Meiyan Jiang, Liyong Yang, Xirong Zhou*

Shanghai Academy of Agricultural Sciences, Shanghai, 201403, *China*

*Corresponding author: zwrape2021@163.com

Supplementary information

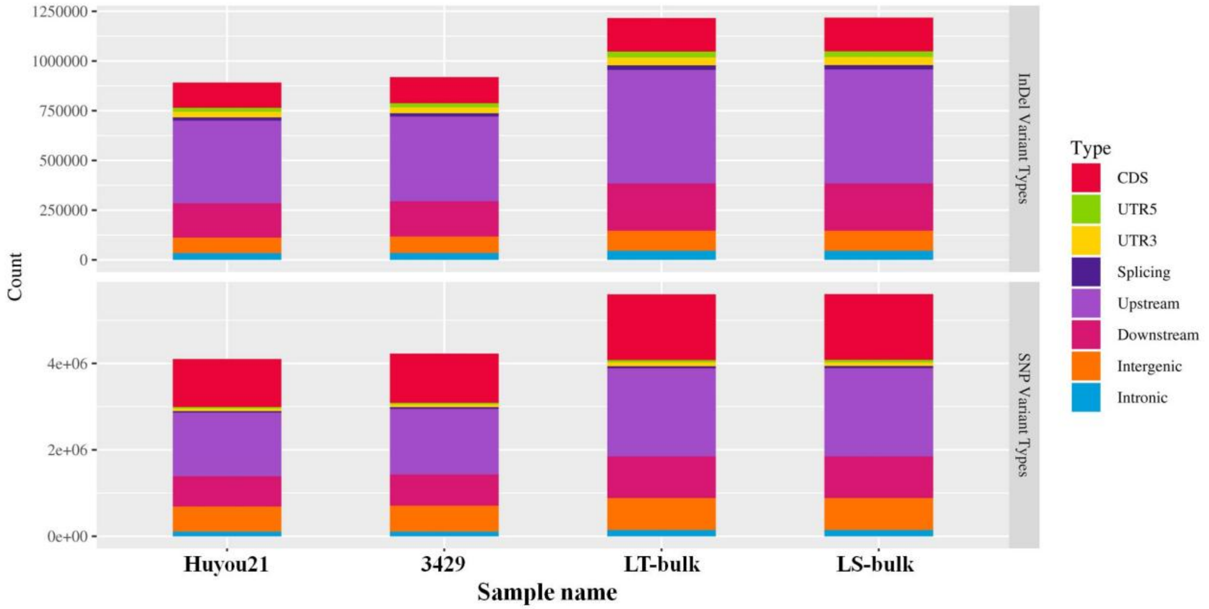

Figure S1. Types of SNP and InDel variants in LT-bulk, ST-bulk and two parents.

Table S1. Rapeseed plants used for LTG mapping and their reactions to low temperature.

Rapeseed material	Tolerant (germination rate>90%)	Susceptible (germination rate≤90%)	Total	Expect ratio	c^2	*P* value
Huyou21	30	0	30	-	-	-
3429	0	30	30	-	-	-
3429/Huyou21 $F_{2:3}$ family	344	230	574	9:7	1.58	0.21

Table S2 Number of SNPs and InDels were identified in two bulks and parents.

Type	LT bulk	LS bulk	Huyou21	3429
Number of SNPs	5,600,575	5,604,534	4,101,701	4,226,553
Number of InDels	1,215,792	1,217,972	892,062	918,828

Table S3 Function variants effects related to the predicted genes in *qLTGA9-1* and *qLTGC1-1*.

QTL	Chr.	Position of mutation site	The base of Huyou21	The base of 3429	GeneID	Mutation type	Functional effect
	A09	44725714	T	A	*chrA09g005475*	upstream_gene_variant	MODIFIER
	A09	44729302	A	T	*chrA09g005475-chrA09g005476*	intergenic_region	MODIFIER
	A09	44748998	TTATATA	T	*chrA09g005478*	downstream_gene_variant	MODIFIER
	A09	44749119	T	TGA	*chrA09g005479*	splice_region_variant&intron_variant	LOW
	A09	44749999	G	A	*chrA09g005479*	synonymous_variant	LOW
	A09	44750018	G	T	*chrA09g005479*	splice_region_variant&intron_variant	LOW
	A09	44750019	C	T	*chrA09g005479*	splice_region_variant&intron_variant	LOW
	A09	44750041	C	T	*chrA09g005478*	downstream_gene_variant	MODIFIER
qLTGA9-1	A09	44750050	T	C	*chrA09g005478*	downstream_gene_variant	MODIFIER
	A09	44750052	A	T	*chrA09g005478*	downstream_gene_variant	MODIFIER
	A09	44750056	A	AT	*chrA09g005478*	downstream_gene_variant	MODIFIER
	A09	44750062	A	T	*chrA09g005478*	downstream_gene_variant	MODIFIER
	A09	44750079	T	A	*chrA09g005478*	downstream_gene_variant	MODIFIER
	A09	44750080	T	A	*chrA09g005478*	downstream_gene_variant	MODIFIER
	A09	44756046	T	G	*chrA09g005480*	upstream_gene_variant	MODIFIER
	A09	44756548	T	TAGACTAAGA AACTTCCACTA	*chrA09g005480*	upstream_gene_variant	MODIFIER

			TG			
A09	44788715	A	T	*chrA09g005489*	upstream_gene_variant	MODIFIER
A09	44789932	C	A	*chrA09g005489*	upstream_gene_variant	MODIFIER
A09	44821604	C	A	*chrA09g005495*	downstream_gene_variant	MODIFIER
A09	44821608	TTG	T	*chrA09g005495*	downstream_gene_variant	MODIFIER
A09	44821618	T	A	*chrA09g005495*	downstream_gene_variant	MODIFIER
A09	44824698	A	G	*chrA09g005495*	synonymous_variant	LOW
A09	44824839	G	A	*chrA09g005494*	downstream_gene_variant	MODIFIER
A09	44837326	T	G	*chrA09g005498*	synonymous_variant	LOW
A09	44842617	C	T	*chrA09g005501*	5_prime_UTR_variant	MODIFIER
A09	44843101	C	A	*chrA09g005501*	missense_variant	MODERATE
A09	44843113	C	T	*chrA09g005501*	missense_variant	MODERATE
A09	44843144	C	T	*chrA09g005501*	synonymous_variant	LOW
A09	44845228	G	A	*chrA09g005502*	missense_variant	MODERATE
A09	44845247	A	C	*chrA09g005503*	upstream_gene_variant	MODIFIER
A09	44845567	AT	A	*chrA09g005503*	upstream_gene_variant	MODIFIER
A09	44849931	T	A	*chrA09g005502*	upstream_gene_variant	MODIFIER
A09	44850179	T	A	*chrA09g005502*	upstream_gene_variant	MODIFIER
A09	44850270	T	C	*chrA09g005502*	upstream_gene_variant	MODIFIER
A09	44850273	G	GGTT	*chrA09g005502*	upstream_gene_variant	MODIFIER
A09	44850274	A	T	*chrA09g005502*	upstream_gene_variant	MODIFIER
A09	44850276	A	T	*chrA09g005502*	upstream_gene_variant	MODIFIER
A09	44854125	G	T	*chrA09g005504*	upstream_gene_variant	MODIFIER
A09	44854354	G	A	*chrA09g005504*	upstream_gene_variant	MODIFIER
A09	44854479	C	T	*chrA09g005504*	upstream_gene_variant	MODIFIER
A09	44855863	CATACATT	C	*chrA09g005504*	upstream_gene_variant	MODIFIER

		TTGAT				
A09	44855883	C	G	*chrA09g005504*	upstream_gene_variant	MODIFIER
A09	44858136	GAACACG AAAGA	G	*chrA09g005504*	upstream_gene_variant	MODIFIER
A09	44858154	C	T	*chrA09g005504*	upstream_gene_variant	MODIFIER
A09	44860421	G	A	*chrA09g005506*	synonymous_variant	LOW
A09	44860544	A	G	*chrA09g005506*	synonymous_variant	LOW
A09	44864396	T	C/A	*chrA09g005507*	missense_variant&splice_region_variant	MODERATE
A09	44864404	G	T	*chrA09g005507*	synonymous_variant	LOW
A09	44865819	T	C	*chrA09g005506*	upstream_gene_variant	MODIFIER
A09	44865838	A	G	*chrA09g005506*	upstream_gene_variant	MODIFIER
A09	44866036	A	AAT	*chrA09g005506*	upstream_gene_variant	MODIFIER
A09	44867603	T	C	*chrA09g005508*	upstream_gene_variant	MODIFIER
A09	44869570	T	A	*chrA09g005509*	synonymous_variant	LOW
A09	44870517	A	ACT	*chrA09g005509*	frameshift_variant	HIGH
A09	44870518	G	GAATGATCA	*chrA09g005509*	frameshift_variant	HIGH
A09	44870576	T	TTC	*chrA09g005509*	frameshift_variant	HIGH
A09	44886229	T	A	*chrA09g005513*	upstream_gene_variant	MODIFIER
A09	44894916	G	A	*chrA09g005514*	3_prime_UTR_variant	MODIFIER
A09	44925087	CTTG	C	*chrA09g005521*	upstream_gene_variant	MODIFIER
A09	44925123	C	A	*chrA09g005521*	upstream_gene_variant	MODIFIER
A09	44929536	C	G	*chrA09g005521*	intron_variant	MODIFIER
A09	44929547	A	ATGATTT	*chrA09g005521*	intron_variant	MODIFIER
A09	44929550	T	TATATTTTTAA TATTATA	*chrA09g005521*	intron_variant	MODIFIER
A09	44943484	A	G	*chrA09g005522*	upstream_gene_variant	MODIFIER

A09	44947180	C	G	*chrA09g005523*	upstream_gene_variant	MODIFIER
A09	44950560	G	A	*chrA09g005523*	synonymous_variant	LOW
A09	44950611	T	C	*chrA09g005523*	synonymous_variant	LOW
A09	44950640	TC	T	*chrA09g005524*	upstream_gene_variant	MODIFIER
A09	44950643	T	G	*chrA09g005524*	upstream_gene_variant	MODIFIER
A09	44950648	T	C	*chrA09g005524*	upstream_gene_variant	MODIFIER
A09	44950651	T	C	*chrA09g005524*	upstream_gene_variant	MODIFIER
A09	44950659	A	AAC	*chrA09g005524*	upstream_gene_variant	MODIFIER
A09	44951412	T	TC	*chrA09g005524*	upstream_gene_variant	MODIFIER
A09	44951414	A	T	*chrA09g005524*	upstream_gene_variant	MODIFIER
A09	44951648	G	A	*chrA09g005523*	missense_variant	MODERATE
A09	44956475	A	T	*chrA09g005523*	downstream_gene_variant	MODIFIER
A09	44958598	G	T	*chrA09g005524*	missense_variant	MODERATE
A09	44958600	C	A	*chrA09g005524*	missense_variant	MODERATE
A09	44958874	G	GT	*chrA09g005524*	splice_region_variant&intron_variant	LOW
A09	44958957	G	A	*chrA09g005524*	missense_variant	MODERATE
A09	44964158	A	ATATTTAATTAGCATTTAGTTATAATCAACCAAGCCTAAAAACT	*chrA09g005526*	splice_region_variant&intron_variant	LOW
A09	44965526	G	A	*chrA09g005525*	upstream_gene_variant	MODIFIER
A09	44965529	C	T	*chrA09g005525*	upstream_gene_variant	MODIFIER
A09	44977881	A	T	*chrA09g005527*	upstream_gene_variant	MODIFIER
A09	44987132	G	T	*chrA09g005528*	upstream_gene_variant	MODIFIER
A09	44987152	A	AT	*chrA09g005528*	upstream_gene_variant	MODIFIER

	A09	44988122	GA	G	*chrA09g005528*	upstream_gene_variant	MODIFIER
	A09	44997815	G	T	*chrA09g005531*	upstream_gene_variant	MODIFIER
	A09	44997816	A	G	*chrA09g005531*	upstream_gene_variant	MODIFIER
	A09	44997817	C	T	*chrA09g005531*	upstream_gene_variant	MODIFIER
	A09	45006449	T	A	*chrA09g005532*	downstream_gene_variant	MODIFIER
	A09	45021586	TC	T	*chrA09g005535*	upstream_gene_variant	MODIFIER
	A09	45023696	G	C	*chrA09g005534*	upstream_gene_variant	MODIFIER
	A09	45028101	A	G	*chrA09g005534*	upstream_gene_variant	MODIFIER
	A09	45055937	T	TCAAAAACAC	*chrA09g005540*	3_prime_UTR_variant	MODIFIER
	A09	45056829	G	A	*chrA09g005540*	5_prime_UTR_variant	MODIFIER
	A09	45057218	T	G	*chrA09g005540*	upstream_gene_variant	MODIFIER
	A09	45057220	G	A	*chrA09g005540*	upstream_gene_variant	MODIFIER
	A09	45060826	A	T	*chrA09g005540*	upstream_gene_variant	MODIFIER
qLTGC1-1	C01	48581745	A	G	*chrC01g004356*	upstream_gene_variant	MODIFIER
	C01	48581783	T	C	*chrC01g004356*	upstream_gene_variant	MODIFIER
	C01	48581789	G	A	*chrC01g004356*	upstream_gene_variant	MODIFIER
	C01	48581797	A	T	*chrC01g004356*	upstream_gene_variant	MODIFIER
	C01	48582452	C	A	*chrC01g004356*	upstream_gene_variant	MODIFIER
	C01	48591406	A	G	*chrC01g004358*	downstream_gene_variant	MODIFIER
	C01	48593949	T	A	*chrC01g004357*	missense_variant	MODERATE
	C01	48593963	G	A	*chrC01g004357*	missense_variant	MODERATE
	C01	48596639	G	T	*chrC01g004359*	missense_variant	MODERATE
	C01	48597324	C	A	*chrC01g004359*	missense_variant	MODERATE
	C01	48597423	A	T	*chrC01g004358*	upstream_gene_variant	MODIFIER
	C01	48597437	T	G	*chrC01g004359*	splice_region_variant&intron_variant	LOW
	C01	48597518	A	C	*chrC01g004358*	upstream_gene_variant	MODIFIER

C01	48598694	A	T	*chrC01g004358*	upstream_gene_variant	MODIFIER
C01	48598695	GA	G	*chrC01g004358*	upstream_gene_variant	MODIFIER
C01	48598697	A	G	*chrC01g004358*	upstream_gene_variant	MODIFIER
C01	48598706	G	C	*chrC01g004358*	upstream_gene_variant	MODIFIER
C01	48598738	A	G	*chrC01g004358*	upstream_gene_variant	MODIFIER
C01	48599149	A	C	*chrC01g004358*	upstream_gene_variant	MODIFIER
C01	48602833	G	A	*chrC01g004360*	synonymous_variant	LOW
C01	48675934	T	A	*chrC01g004367*	upstream_gene_variant	MODIFIER
C01	48675963	C	T	*chrC01g004367*	upstream_gene_variant	MODIFIER
C01	48683135	T	T/C	*chrC01g004369-chrC01g004370*	intergenic_region	MODIFIER
C01	48683136	G	G/A	*chrC01g004369-chrC01g004370*	intergenic_region	MODIFIER
C01	48688398	C	A	*chrC01g004370*	upstream_gene_variant	MODIFIER
C01	48690224	GT	G	*chrC01g004370*	upstream_gene_variant	MODIFIER
C01	48691182	G	GT	*chrC01g004370*	upstream_gene_variant	MODIFIER
C01	48691183	A	C	*chrC01g004370*	upstream_gene_variant	MODIFIER
C01	48691240	A	G	*chrC01g004370*	upstream_gene_variant	MODIFIER
C01	48693198	C	A	*chrC01g004370*	downstream_gene_variant	MODIFIER
C01	48693199	T	G	*chrC01g004370*	downstream_gene_variant	MODIFIER
C01	48694436	T	A	*chrC01g004370*	downstream_gene_variant	MODIFIER
C01	48694443	C	A	*chrC01g004370*	downstream_gene_variant	MODIFIER
C01	48694459	T	TA	*chrC01g004370*	downstream_gene_variant	MODIFIER
C01	48694552	G	A	*chrC01g004370*	downstream_gene_variant	MODIFIER
C01	48698300	A	T	*chrC01g004371*	upstream_gene_variant	MODIFIER
C01	48698301	A	T	*chrC01g004371*	upstream_gene_variant	MODIFIER
C01	48702415	A	G	*chrC01g004371*	upstream_gene_variant	MODIFIER

C01	48706515	A	T	*chrC01g004373*	upstream_gene_variant	MODIFIER
C01	48710527	C	A	*chrC01g004372*	upstream_gene_variant	MODIFIER
C01	48722392	C	A	*chrC01g004373-chrC01g004374*	intergenic_region	MODIFIER
C01	48724598	A	G	*chrC01g004373-chrC01g004374*	intergenic_region	MODIFIER
C01	48726471	C	T	*chrC01g004373-chrC01g004374*	intergenic_region	MODIFIER
C01	48726500	T	G	*chrC01g004373-chrC01g004374*	intergenic_region	MODIFIER
C01	48726782	A	G	*chrC01g004373-chrC01g004374*	intergenic_region	MODIFIER
C01	48726829	T	G	*chrC01g004373-chrC01g004374*	intergenic_region	MODIFIER
C01	48726834	G	A	*chrC01g004373-chrC01g004374*	intergenic_region	MODIFIER
C01	48726837	G	A	*chrC01g004373-chrC01g004374*	intergenic_region	MODIFIER
C01	48726870	G	A	*chrC01g004373-chrC01g004374*	intergenic_region	MODIFIER
C01	48728559	A	T	*chrC01g004373-chrC01g004374*	intergenic_region	MODIFIER
C01	48728560	A	C	*chrC01g004373-chrC01g004374*	intergenic_region	MODIFIER
C01	48728573	G	A	*chrC01g004373-chrC01g004374*	intergenic_region	MODIFIER
C01	48729972	C	A	*chrC01g004373-chrC01g004374*	intergenic_region	MODIFIER
C01	48730026	G	C	*chrC01g004373-chrC01g004374*	intergenic_region	MODIFIER
C01	48730054	C	T	*chrC01g004373-chrC01g004374*	intergenic_region	MODIFIER
C01	48730062	T	C	*chrC01g004373-chrC01g004374*	intergenic_region	MODIFIER
C01	48730080	C	A	*chrC01g004373-chrC01g004374*	intergenic_region	MODIFIER
C01	48730728	C	G	*chrC01g004373-chrC01g004374*	intergenic_region	MODIFIER
C01	48731414	A	C	*chrC01g004373-chrC01g004374*	intergenic_region	MODIFIER
C01	48731425	C	T	*chrC01g004373-chrC01g004374*	intergenic_region	MODIFIER
C01	48733087	A	G	*chrC01g004373-chrC01g004374*	intergenic_region	MODIFIER
C01	48733099	C	T	*chrC01g004373-chrC01g004374*	intergenic_region	MODIFIER
C01	48733288	G	A	*chrC01g004373-chrC01g004374*	intergenic_region	MODIFIER

C01	48733313	T	C	*chrC01g004373-chrC01g004374*	intergenic_region	MODIFIER
C01	48737898	C	T	*chrC01g004374*	upstream_gene_variant	MODIFIER
C01	48738369	T	C	*chrC01g004374*	upstream_gene_variant	MODIFIER
C01	48739102	C	T	*chrC01g004374*	upstream_gene_variant	MODIFIER
C01	48776749	TGGTTCTCGAAATGTTAAGACATCA	T	*chrC01g004380*	upstream_gene_variant	MODIFIER
C01	48964805	A	C	*chrC01g004389*	upstream_gene_variant	MODIFIER
C01	48980685	C	T	*chrC01g004390*	upstream_gene_variant	MODIFIER
C01	48985814	A	AGGGTTAGGTGAGATTGGGTTTTCGAACTTATCGTCATTAGTTTTTTATTCT	*chrC01g004393*	upstream_gene_variant	MODIFIER
C01	48990922	C	CT	*chrC01g004395*	upstream_gene_variant	MODIFIER
C01	48990960	G	A	*chrC01g004395*	upstream_gene_variant	MODIFIER
C01	48991068	CT	C	*chrC01g004395*	upstream_gene_variant	MODIFIER
C01	48997407	T	C	*chrC01g004395-chrC01g004396*	intergenic_region	MODIFIER
C01	48997580	T	C	*chrC01g004395-chrC01g004396*	intergenic_region	MODIFIER
C01	49007058	G	T	*chrC01g004396*	intron_variant	MODIFIER
C01	49007679	A	C	*chrC01g004396*	intron_variant	MODIFIER
C01	49008909	A	C	*chrC01g004396*	downstream_gene_variant	MODIFIER
C01	49014161	T	C	*chrC01g004396-chrC01g004397*	intergenic_region	MODIFIER
C01	49015675	A	C	*chrC01g004396-chrC01g004397*	intergenic_region	MODIFIER
C01	49016614	G	T	*chrC01g004396-chrC01g004397*	intergenic_region	MODIFIER

C01	49018372	TG	T	*chrC01g004396-chrC01g004397*	intergenic_region	MODIFIER
C01	49020692	G	A	*chrC01g004396-chrC01g004397*	intergenic_region	MODIFIER
C01	49020885	T	A	*chrC01g004396-chrC01g004397*	intergenic_region	MODIFIER
C01	49021105	T	A	*chrC01g004396-chrC01g004397*	intergenic_region	MODIFIER
C01	49024325	AT	ATTTT	*chrC01g004397*	upstream_gene_variant	MODIFIER
C01	49026445	C	T	*chrC01g004397*	upstream_gene_variant	MODIFIER
C01	49038207	G	A	*chrC01g004397-chrC01g004398*	intergenic_region	MODIFIER
C01	49038859	T	C	*chrC01g004397-chrC01g004398*	intergenic_region	MODIFIER
C01	49047923	C	T	*chrC01g004397-chrC01g004398*	intergenic_region	MODIFIER
C01	49050695	A	G	*chrC01g004397-chrC01g004398*	intergenic_region	MODIFIER
C01	49050766	T	T/A	*chrC01g004397-chrC01g004398*	intergenic_region	MODIFIER
C01	49052162	GT	G	*chrC01g004398*	upstream_gene_variant	MODIFIER
C01	49052195	A	G	*chrC01g004398*	upstream_gene_variant	MODIFIER
C01	49055222	AT	A	*chrC01g004398*	upstream_gene_variant	MODIFIER
C01	49060711	A	G	*chrC01g004398*	downstream_gene_variant	MODIFIER
C01	49068422	AT	A	*chrC01g004399*	upstream_gene_variant	MODIFIER
C01	49068428	G	A	*chrC01g004399*	upstream_gene_variant	MODIFIER
C01	49077473	A	G	*chrC01g004400*	upstream_gene_variant	MODIFIER
C01	49077489	C	T	*chrC01g004400*	upstream_gene_variant	MODIFIER
C01	49082732	C	T	*chrC01g004400*	missense_variant	MODERATE
C01	49082807	G	A	*chrC01g004400*	downstream_gene_variant	MODIFIER
C01	49084249	T	C	*chrC01g004400*	downstream_gene_variant	MODIFIER
C01	49084390	T	TA	*chrC01g004400*	downstream_gene_variant	MODIFIER
C01	49084409	A	G	*chrC01g004400*	downstream_gene_variant	MODIFIER
C01	49092369	ATTTT	A	*chrC01g004400-chrC01g004401*	intergenic_region	MODIFIER

C01	49092376	T	TAAAA	*chrC01g004400-chrC01g004401*	intergenic_region	MODIFIER
C01	49092398	G	C	*chrC01g004400-chrC01g004401*	intergenic_region	MODIFIER
C01	49093936	T	C	*chrC01g004400-chrC01g004401*	intergenic_region	MODIFIER
C01	49093985	C	A	*chrC01g004400-chrC01g004401*	intergenic_region	MODIFIER
C01	49094046	G	C	*chrC01g004400-chrC01g004401*	intergenic_region	MODIFIER
C01	49094886	C	A	*chrC01g004400-chrC01g004401*	intergenic_region	MODIFIER
C01	49099328	T	G	*chrC01g004401*	upstream_gene_variant	MODIFIER
C01	49099800	C	T	*chrC01g004401*	upstream_gene_variant	MODIFIER
C01	49100826	A	G	*chrC01g004401*	upstream_gene_variant	MODIFIER
C01	49102292	G	A	*chrC01g004401*	upstream_gene_variant	MODIFIER
C01	49102864	A	C	*chrC01g004401*	upstream_gene_variant	MODIFIER
C01	49104053	T	TA	*chrC01g004401*	downstream_gene_variant	MODIFIER
C01	49110765	G	A	*chrC01g004401-chrC01g004402*	intergenic_region	MODIFIER
C01	49112639	T	A	*chrC01g004401-chrC01g004402*	intergenic_region	MODIFIER
C01	49119847	G	T	*chrC01g004401-chrC01g004402*	intergenic_region	MODIFIER
C01	49121357	C	T	*chrC01g004401-chrC01g004402*	intergenic_region	MODIFIER
C01	49121877	A	G	*chrC01g004402*	upstream_gene_variant	MODIFIER
C01	49127149	C	T	*chrC01g004402*	downstream_gene_variant	MODIFIER
C01	49144001	C	A	*chrC01g004403*	upstream_gene_variant	MODIFIER
C01	49147710	G	A	*chrC01g004403-chrC01g004404*	intergenic_region	MODIFIER
C01	49151537	AT	A	*chrC01g004403-chrC01g004404*	intergenic_region	MODIFIER
C01	49152352	G	A	*chrC01g004403-chrC01g004404*	intergenic_region	MODIFIER
C01	49166356	T	A	*chrC01g004405*	missense_variant	MODERATE
C01	49166516	G	T	*chrC01g004405*	missense_variant	MODERATE
C01	49166868	C	T	*chrC01g004405*	upstream_gene_variant	MODIFIER

C01	49166980	T	TGA	*chrC01g004405*	upstream_gene_variant	MODIFIER
C01	49167446	C	G	*chrC01g004405*	upstream_gene_variant	MODIFIER
C01	49171172	C	T	*chrC01g004405*	upstream_gene_variant	MODIFIER
C01	49171195	T	G	*chrC01g004405*	upstream_gene_variant	MODIFIER
C01	49175650	A	G	*chrC01g004405-chrC01g004406*	intergenic_region	MODIFIER
C01	49176074	A	G	*chrC01g004405-chrC01g004406*	intergenic_region	MODIFIER
C01	49176465	T	C	*chrC01g004405-chrC01g004406*	intergenic_region	MODIFIER
C01	49185350	A/G	G	*chrC01g004405-chrC01g004406*	intergenic_region	MODIFIER
C01	49185677	A	G	*chrC01g004405-chrC01g004406*	intergenic_region	MODIFIER
C01	49185727	T	T/C	*chrC01g004405-chrC01g004406*	intergenic_region	MODIFIER
C01	49188139	G	A	*chrC01g004405-chrC01g004406*	intergenic_region	MODIFIER
C01	49193585	T	G	*chrC01g004406*	missense_variant	MODERATE
C01	49200554	A	G	*chrC01g004407*	synonymous_variant	LOW
C01	49200966	C	C/T	*chrC01g004407*	5_prime_UTR_variant	MODIFIER
C01	49201560	T	G	*chrC01g004407*	upstream_gene_variant	MODIFIER
C01	49201564	T	C	*chrC01g004407*	upstream_gene_variant	MODIFIER
C01	49202410	T	A	*chrC01g004407*	upstream_gene_variant	MODIFIER
C01	49207475	C	T	*chrC01g004408*	upstream_gene_variant	MODIFIER
C01	49207491	C	T	*chrC01g004408*	upstream_gene_variant	MODIFIER
C01	49207528	C	G	*chrC01g004408*	upstream_gene_variant	MODIFIER
C01	49212286	A	G	*chrC01g004409*	upstream_gene_variant	MODIFIER
C01	49217623	C	A	*chrC01g004409*	downstream_gene_variant	MODIFIER
C01	49228519	A	C	*chrC01g004409-chrC01g004410*	intergenic_region	MODIFIER
C01	49228566	A	G	*chrC01g004409-chrC01g004410*	intergenic_region	MODIFIER
C01	49228576	G	T	*chrC01g004409-chrC01g004410*	intergenic_region	MODIFIER

C01	49228588	A	G	*chrC01g004409-chrC01g004410*	intergenic_region	MODIFIER
C01	49228821	G	A	*chrC01g004409-chrC01g004410*	intergenic_region	MODIFIER
C01	49229190	A	G	*chrC01g004409-chrC01g004410*	intergenic_region	MODIFIER
C01	49229244	CA	C	*chrC01g004409-chrC01g004410*	intergenic_region	MODIFIER
C01	49229259	T	C	*chrC01g004409-chrC01g004410*	intergenic_region	MODIFIER
C01	49230476	C	T	*chrC01g004409-chrC01g004410*	intergenic_region	MODIFIER
C01	49239332	G	A	*chrC01g004409-chrC01g004410*	intergenic_region	MODIFIER
C01	49239559	A	G	*chrC01g004409-chrC01g004410*	intergenic_region	MODIFIER
C01	49239819	A	T	*chrC01g004409-chrC01g004410*	intergenic_region	MODIFIER
C01	49257001	CT	C	*chrC01g004410*	upstream_gene_variant	MODIFIER
C01	49257061	G	C	*chrC01g004410*	upstream_gene_variant	MODIFIER
C01	49274139	A	C	*chrC01g004411-chrC01g004412*	intergenic_region	MODIFIER
C01	49274193	A	T	*chrC01g004411-chrC01g004412*	intergenic_region	MODIFIER
C01	49274204	T	TATAC	*chrC01g004411-chrC01g004412*	intergenic_region	MODIFIER
C01	49274232	A	T	*chrC01g004411-chrC01g004412*	intergenic_region	MODIFIER
C01	49274244	T	A	*chrC01g004411-chrC01g004412*	intergenic_region	MODIFIER
C01	49274245	T	A	*chrC01g004411-chrC01g004412*	intergenic_region	MODIFIER
C01	49274252	A	AG	*chrC01g004411-chrC01g004412*	intergenic_region	MODIFIER
C01	49310544	T	G	*chrC01g004414*	upstream_gene_variant	MODIFIER
C01	49310550	T	A	*chrC01g004414*	upstream_gene_variant	MODIFIER
C01	49310636	G	A	*chrC01g004414*	upstream_gene_variant	MODIFIER
C01	49310647	T	C	*chrC01g004414*	upstream_gene_variant	MODIFIER
C01	49312896	A	G	*chrC01g004414*	downstream_gene_variant	MODIFIER
C01	49312936	T	G	*chrC01g004414*	downstream_gene_variant	MODIFIER
C01	49312937	A	T	*chrC01g004414*	downstream_gene_variant	MODIFIER

C01	49313012	G	C	*chrC01g004414*	downstream_gene_variant	MODIFIER
C01	49313019	T	TAGTAC	*chrC01g004414*	downstream_gene_variant	MODIFIER
C01	49313159	C	T	*chrC01g004414*	downstream_gene_variant	MODIFIER
C01	49313318	T	TTATA	*chrC01g004414*	downstream_gene_variant	MODIFIER
C01	49313383	C	CA	*chrC01g004414*	downstream_gene_variant	MODIFIER
C01	49313431	T	A	*chrC01g004414*	downstream_gene_variant	MODIFIER
